# Concerning Old Age Pensions

**Published:** 1895-07-13

**Authors:** 


### Modern Sociology.

### CONCERNING OLD AGE PENSIONS.

A correspondent asks us how an old age pension scheme could be brought before the country. We might reasonably decline to formulate any plans on a subject which is now occupying the attention of experienced politicians, but we can at least indicate the limits within which such a scheme should work, and the means by which, in our opinion, it might be secured.

First, it is necessary to define what we mean by the word "pension," for some take it as representing a free gift out of the State funds, and others as a system of insurance. We accept the latter rendering. The pensions are to be paid from the contributions of the pensioners, after these have accumulated in the hands of a State department composed—whatever decorative figurehead may pretend to rule it—of experienced actuaries who shall be permanent officials. The official who goes in and out with successive Governments would be almost useless for such work as this. In the second place, the contribution must be universal and compulsory. The highest peer, the richest millionaire in the country, should pay like the ploughman and the artisan. Thirdly, if the contributor dies before attaining the pension age, the policy must be forfeited. Without these conditions a reasonable pension could not be given without too heavily taxing the working classes. It has been contended that people will not contribute to a fund from which they expect to receive no benefit. Is this true? We pay the school-rate, yet many of us do not send our children to Board schools. Most of us pay fire insurances which will lapse with our death, and all the time hope that we may never need to benefit by them. An actuary could estimate, from the returns of the Registrar-General and those of the income-tax commissioners, the amount of unclaimed money which would thus fall into the general fund. It must be remembered, however, that though the creation of old age pensions would largely diminish pauperism, and would almost, if not quite, get rid of the pauper who receives outdoor relief, the pensions would be claimed by many others than those who are now paupers. There should be no stigma attached to the pension. It is an investment, not a charity, though, perhaps, more remunerative than a "safe" investment usually is.

From this point of view we regard the talk about "the deserving poor" as unfortunate. If an old age pension scheme is ever to become a national institution, there must be no question of poverty, there must be no question of desert. If Lord Rothschild or the Duke of Westminster chooses to claim his national pension, let him be free to do so. If Mr. William Sikes, having got off with some years of penal servitude instead of the hanging he deserved for that Nancy business, comes forward to demand his money, he shall not be refused. All that he must do is to prove his identity and that he has paid his premiums. As soon as any question of fitness arises the pension scheme will degenerate into such a political bribe as has recently disgraced the Government of the United States, or into a Sunday-school prize to be gained by the favour of parsons and great ladies. It is one of the objections to any scheme based on pure donation and selection that Uriah Heep will so distinctly have the best of it.

If, however, the pensions are to be really old age insurances, when and how are the premiums to be collected? When? While the insurer is between the ages of, say, fifteen and twenty-five. The wage-earning classes are, as a rule, out at work at fifteen. Their wages are small—often do not pay the expense of keeping them—but we believe that many parents among the working classes would willingly give part of their children's earnings towards paying their pension contribution. Failing this, the young men and women of the working class are, as a rule, entirely independent after twenty—some of them marry at eighteen—and if the pension contribution made them postpone their marriage for a few years it would do them no ill turn. Between twenty and twenty-five, however, many are earning their maximum wage, while their expenses are at their smallest. Most are unmarried, and, with regard to the others, their family expenses are less than they will probably be later. We would suggest, however, that policies should be issued at higher premiums up to a greater age; all premiums to be calculated according to the ordinary principles of life insurance, and due allowance being made for the possible increment from unclaimed money. It should also be optional in what proportions the premiums should be paid, as at one time it might be easier than another to deduct something from wages.

As to the method of collecting, there will certainly be difficulties, but not necessarily of an insuperable nature. Employers of labour are in the habit of making deductions from wages for medical attendance, and, when they provide houses, for rent. This principle could surely be carried further, the employé signing a document empowering the employer to deduct a certain sum a week, or a certain percentage in the pound, from his wages, and receiving a receipt for each such deduction. The weekly receipts might be exchanged for quarterly and yearly ones at the expiry of such periods, to do away with the multiplication of documents, and the employer should periodically forward the money deducted, and the names and respective amounts paid, to the Government Pension Bureau. When the amount necessary to secure a pension has been paid, a policy should be given to the contributor. It may be objected that in the changes and chances of a working man's life the policy might be lost, but it is wonderful how seldom people lose things they greatly care to keep. How many women lose their "marriage lines"? How many men lose the book in which their contributions to a friendly society are recorded? In the event of a policy being lost, the onus of proving his claim must rest with the loser. The selling of a pension should be made illegal, and the seller be treated with the full rigour of the poor law, for a poor law would still be required for those who managed to evade taking out a pension policy. But as these would be more assuredly the idle, improvident, and disreputable than paupers are at present, there would be less objection to stern discipline and plain fare than there is at present. We have not ventured on estimates of the amount of pension desirable, nor the premium necessary to assure it—that is for trained actuaries to do, but we have indicated the lines on which we think a pension scheme might feasibly be carried out, which should be helpful and not pauperising.

